# Digital Nudging for Online Food Choices

**DOI:** 10.3389/fpsyg.2021.729589

**Published:** 2021-12-20

**Authors:** Mathias Jesse, Dietmar Jannach, Bartosz Gula

**Affiliations:** ^1^Department of AI and Cybersecurity, University of Klagenfurt, Klagenfurt, Austria; ^2^Department of Psychology, University of Klagenfurt, Klagenfurt, Austria

**Keywords:** digital nudging, health, food, consumer behavior, choice satisfaction

## Abstract

When people search for what to cook for the day, they increasingly use online recipe sites to find inspiration. Such recipe sites often show popular recipes to make it easier to find a suitable choice. However, these popular recipes are not always the healthiest options and can promote an unhealthy lifestyle. Our goal is to understand to what extent it is possible to steer the food selection of people through digital nudging. While nudges have been shown to affect humans' behavior regarding food choices in the physical world, there is little research on the impact of nudges on online food choices. Specifically, it is unclear how different nudges impact *(i)* the behavior of people, *(ii)* the time they need to make a decision, and *(iii)* their satisfaction and confidence with their selection. We investigate the effects of highlighting, defaults, social information, and warnings on the decision-making of online users through two consecutive user studies. Our results show that a hybrid nudge, which both involves setting a default and adding social information, significantly increases the likelihood that a nudged item is selected. Moreover, it may help decreasing the required decision time for participants while having no negative effects on the participant's satisfaction and confidence. Overall, our work provides evidence that nudges can be effective in this domain, but also that the type of a digital nudge matters. Therefore, different nudges should be evaluated in practical applications.

## 1. Introduction

Obesity and unhealthy eating behavior are becoming more problematic nowadays. Trends identified by the World Health Organization (WHO, 2020) show that the proportion of overweight children and adults has increased in recent years. Specifically, over 1.9 billion adults and 340 million children and adolescents were overweight in 2016. Research shows that obese people have a higher risk of noncommunicable diseases such as cardiovascular diseases, diabetes, musculoskeletal disorders, and cancer (WHO, 2020). If people are overweight in their childhood, they are at risk of being overweight in their adult years, premature death, and other risks in the future. Therefore, it is necessary to assist people, particularly younger ones, in making healthier choices regarding their daily intake.

When it comes to finding inspiration for new recipes to try out, people increasingly use online recipe sites like *allrecipes.com*. However, when online recipe sites suggest popular recipes, these might not always be the healthiest ones (Elsweiler et al., 2017). Moreover, Trattner and Elsweiler (2017) showed that unhealthy recipes receive the highest ratings, most comments, are bookmarked most often, and get the most attention overall. Typically, these unhealthy recipes shared online are excessive in saturated fat and sodium. Furthermore, online recipes are the least healthy ones overall compared to super-market-ready meals and recipes developed by TV chefs (Trattner and Elsweiler, 2017). We also see a connection between interactions with recipes (i.e., ratings, comments, or bookmarks) and the resulting consumption behavior. One example of this is the study conducted by Trattner et al. (2017). The authors investigated the relationship between bookmarked recipes on *allrecipes.com* and the resulting food and health-related issues. They showed that if users bookmarked recipes that contained a high amount of fat and sugar, these users turned out to have a higher chance of obesity. In a study conducted by Said and Bellogín (2014), the authors compared users' interaction patterns with recipes on *allrecipes.com* between geographical areas known to have relatively good or poor health (i.e., higher obesity rates). They identified users from geographical regions known for bad health solely through their interactions with online recipes. The authors found users from that region by comparing the usage of certain ingredients (e.g., garlic, olive oil, or dairy products) in these recipes. For example, geographical regions known for good health use garlic more often than regions known for bad health. Another study conducted by De Choudhury et al. (2016), the authors investigated the interaction of users with online recipes in rural areas and urban cities, which they referred to as food-deserts. These food-deserts were characterized for having poor access to healthy and affordable food, and the people living in these generally suffer from poor diet and diet-related health outcomes. They found that based on the interactions with dishes on social media, users from these food-deserts consumed food higher (around 5–17%) in fat, cholesterol, and sugar compared to non-food desert areas.

While there are numerous methods to positively influence people's nutritional intake (e.g., different medical treatments and diets), many of them can require significant efforts and resources to be broadly successful (Avenell et al., 2004; Arno and Thomas, 2016; Vecchio and Cavallo, 2019). Usually, these methods work only in small-scale communities that need to be isolated and tracked, which makes these methods difficult to scale or almost impractical (Arno and Thomas, 2016). Because of these practical limitations, researchers started to investigate alternative ways of influencing decisions. *Nudges* are an example of such a comparably light-weight and less costly alternative, which may consist of, for example, highlighting the healthier option in a given decision situation (Evers et al., 2018).

Thaler and Sunstein (2008) coined the term *nudging*, and defined it as any aspect of the choice architecture that changes people's behavior. By design, nudges must be in the interest of the person being influenced, not change the economic incentives, not forbid any options, and have predictable outcomes. The concept of nudges was later transferred into the online world, often termed as digital nudging (Weinmann et al., 2016; Meske and Potthoff, 2017; Mirsch et al., 2017). Digital nudges influence people's behavior mainly through changes to the user interface (UI), and such nudges have been tested in various domains like health (Marcano-Olivier et al., 2020), transit (Bothos et al., 2016), or e-commerce (Esposito et al., 2017). In general, digital nudging, as well as traditional offline nudging, has shown to be a promising means for influencing people's decisions.

Assisting people in “offline” decision scenarios to make healthy food choices is relatively well understood, and various studies found that nudges successfully influenced the selection of items in such real-life scenarios. Arno and Thomas (2016), for example, reported in their systematic review that in one study nudges had a positive effect on healthy eating behavior, leading to 15.3% healthier dietary choices. Other studies aimed to understand which nudges for healthy eating are the most effective ones. Cadario and Chandon (2020) performed a meta-analysis of the existing literature. Overall, they found that nudges are more effective when they aim at reducing unhealthy eating (in contrast to aiming to reduce the total intake or increasing healthy eating). Most examined nudges successfully influenced decisions and led to a reduced intake of sugar and overall energy. Another study by Bergeron et al. (2019) investigated the effects of *defaults* on selecting dishes in a restaurant. In particular, the researchers examined alternative menu layouts, with one dessert preparation method already pre-selected. Their findings suggested that modifying the design of the menu in that way can help promote healthier choices.

While *digital* nudging has been explored in different areas already, research on digital nudges in online food choices is still scarce (Berger et al., 2020). Moreover, existing research often focuses only on a small set of nudges—most importantly setting defaults or social information—short-term effects, and they sometimes rely on very specific ways of implementing the nudges. Elsweiler et al. (2017), for example, showed that the choice of the pictures of the recipes could influence a user's final selection. In their case, they steered study participants toward more health-oriented choices (e.g., ones containing less fat). Technically, Elsweiler et al. (2017) applied machine learning methods to predict the characteristics of pictures that would lead to an increased selection rate for the corresponding recipe. They then selected the pictures in a way that favored the choice of recipes with less fat. Another study conducted by Starke et al. (2021) also focused on the use of pictures alongside recipes. Their findings suggest that using visually attractive pictures may increase the selection of healthier recipes. Furthermore, they showed that recipes at the top of the recommendation list have a higher chance of being selected than recipes further down the recommendation list. In a different study, Hoenink et al. (2020) investigated the effects of digital nudges and of showing additional pricing-related information on the consumers' purchasing behavior in an online grocery store. They found that combining a salience-enhancing nudge with the provision of pricing-related information increased the sales—and thus consumption—of healthy items. However, applying the nudge and showing the pricing-related information in isolation did not lead to a significant effect.

A number of related studies led to the similar observation that individual digital nudges are not always effective, i.e., they sometimes do *not* exert strong effects on the decision behavior of users. One example is the work by Forwood et al. (2015). In their study, online customers were offered to swap an unhealthy item in their shopping cart for a healthier one at different points of the shopping process. In the end, they concluded that offering such food swaps as nudges only had limited potential of influencing a customer toward healthier options. Also, Lee et al. (2011) and Berger et al. (2020) identified situations where digital nudges did not always work as expected, both of them in the context of online grocery stores. Berger et al. (2020) investigated to what extent three types of nudges influence the decisions of the online shoppers: *(i)* setting a default; *(ii)* simplifying the decision process by providing summarizing information about the ecological sustainability of each product, *(iii)* providing information about other people's choice behavior. They found that setting defaults and simplification worked in their study, but providing social information as a nudge did *not* significantly influence the selection of the items. The default nudge was also effective in the study by Lee et al. (2011), also when combined with a highlighting nudge. The authors additionally tried out two more nudges, one providing additional information about an item and one emphasizing the advantages of one option compared to another. These two additional nudges were, however, not effective.

Looking at the discussed previous work, not all types of digital nudges might be similarly effective in the food domain. Sometimes only a combination of nudging principles may lead to the desired impact on people's choices. A recent review on food choice architecture and healthy eating behavior concludes that evidence on the combined effect of multiple nudges is scarce (Ensaff, 2021). Moreover, previous studies mostly focused solely on the immediate decision outcome, i.e., which option was finally chosen, and less on factors such as participants' experience of the decision process. Aspects such as decision effort or satisfaction with a decision might be essential to ensure a more sustainable impact of nudges. Summarizing, looking at previous studies, it sometimes remains unclear how different types of nudges impact *(i)* the behavior of people, *(ii)* the time they need to make a decision, and *(iii)* their satisfaction and confidence with their selection.

Our research aims to close some of these research gaps regarding our understanding of nudging in online environments. Specifically, we investigate to what extent nudges are a suitable means to influence the food choice behavior of online users and to what extent the type of nudges matters. Moreover, we analyze the potential impact of nudging on the user's decision effort and confidence and satisfaction with their decisions. We conducted two consecutive studies (*N* = 206) to answer these research questions. In these studies, participants were tasked to select three recipes to try out from five different categories. In the treatment group of the first study, different nudges were implemented in the user interface for making the choices. The first study showed that a “hybrid” nudge, consisting of a default selection and the provision of social information, had a significant effect on the choices of the participants in the treatment group. In the second study, we could validate that the effectiveness of the hybrid nudge is not limited to a certain category of food. Additionally, we found a positive effect of the nudge on the decision time without any negative effect on decision satisfaction. Furthermore, we observed that female and male participants seem to experience nudges differently regarding perceived decision difficulty and satisfaction.

Our work has important practical implications for the implementation of digital nudges. First, we find that the selection of a nudge has to be done with care. Design choices matter in this regard, and also combining nudges may be more effective than using individual nudges in isolation. Second, nudges may help decrease the overall time online users need to make decisions, leading to higher decision efficiency. Third, we observed differences between how male and female participants experience nudges. Therefore, it may be advisable to consider such aspects when designing or selecting a nudge for a specific target audience.

The paper is organized as follows. In section 2, we give a first overview of the two studies. Sections 3, 4 then provide more detail regarding the first and second study, respectively, both in terms of their design and outcomes. Section 5 provides additional analyses covering both studies, e.g., regarding decision times and the provided qualitative feedback. Section 6, finally, discusses the implications of our findings.

## 2. Overview of Studies

We conducted two consecutive studies, which we term *Study-1* and *Study-2* from here on. In both studies, participants were tasked to select recipes that they would like to try out, using a web application that was developed for the purpose of the study. The participants started with their tasks after reading the instructions and after providing informed consent. The overall task setup was as follows.

As a *first step*, participants were asked to select three out of five food categories in which they would like to make their recipe choices. The categories were Vegetarian, Pasta, Fish, Sandwiches, and Desserts. This selection of categories should foster that participants chose recipes in categories they like. In the *second step*, participants had to choose one out of six recipes in each of the selected three categories. The selection was made sequentially, with individual screens for each category. After participants had chosen one of the categories, they were asked to describe the motivation for choosing the recipe. Furthermore, participants were instructed to give feedback on the perceived choice difficulty, their decision satisfaction, and their decision confidence after each choice. After providing this feedback, they were forwarded to the next category. In the *third step*, after selecting one recipe in all three categories, participants filled out a questionnaire on their cooking abilities, health orientation, age, and gender.

In *Study-1*, participants were randomly assigned to either the treatment group or the control group, and participants of both groups completed the three steps described above. For the participants in the treatment group, however, the UI of the screens on which they had to choose one of the six recipes was extended with a nudge. Specifically, for each recipe category, one particular nudge was implemented. The nudges were nested within the different recipe categories to create a more diversified task for participants. In contrast to a crossed design, where each nudge would be found in each recipe category, the nested design assigned a nudge to one food category. Table 1 shows which nudge was used for each category: participants were shown a highlighted recipe in the Vegetarian category, a default recipe was set for Pasta recipes, and social information was presented for Fish dishes. The category of Sandwiches used the hybrid nudge. With this nested design, *Study-1* aimed to explore the effectiveness of nudges across different food domains.

**Table 1:** Nudges implemented in different categories.

**Food category**	**Nudge in** ***Study-1***	**Nudge in** ***Study-2***
Vegetarian	Highlighting	Hybrid nudge
Sandwiches	Hybrid	Hybrid nudge
Pasta	Default	Hybrid nudge
Fish	Social nudge	Hybrid nudge
Desserts	Warning	Warning

Which nudge was used in which recipe category was determined randomly before the experiment and kept static throughout *Study-1* and *Study-2*. In the treatment group of both studies, we showed the corresponding nudge for each category for the second recipe in the list. We deliberately did not nudge the first recipe, as it would have been more challenging to separate the effect of nudge and the positional effect at the very first position. As shown in other works on digital nudging, position biases can exist so that items at the top position in a list often receive more attention than other items (Meske and Potthoff, 2017; Mirsch et al., 2017). The order of the categories in the study was also determined randomly in advance and kept constant throughout the experiment.

*Study-2*, which aimed to validate the effectiveness of the best-performing nudge from *Study-1* across categories, was identical to *Study-1* except that in this treatment group we used the same nudge for all recipe categories, see Table 1. The only exception is the *warning nudge*, which was used for the Dessert category both in *Study-1* and *Study-2*. The reason for keeping this constant will become clear later.

## 3. Main Results: Study-1

### 3.1. Materials and Methods

#### 3.1.1. Study Design

Participants in the treatment group in *Study-1*, as described above, were those who interacted with a choice interface that included nudges of different types, depending on the recipe categories chosen in the first step. *Study-1* is an exploratory study whose aim was to investigate the effectiveness of different nudges. The nudges that we considered were selected by analyzing current works that focused on digital nudging in food-related applications. Specifically, we found that researchers had previously explored the use of the *default* nudge, the *highlighting* nudge, the *social* nudge, and the *warning* nudge (Sunstein, 2014; Weinmann et al., 2016; Theocharous et al., 2019).

**Figure 1:**
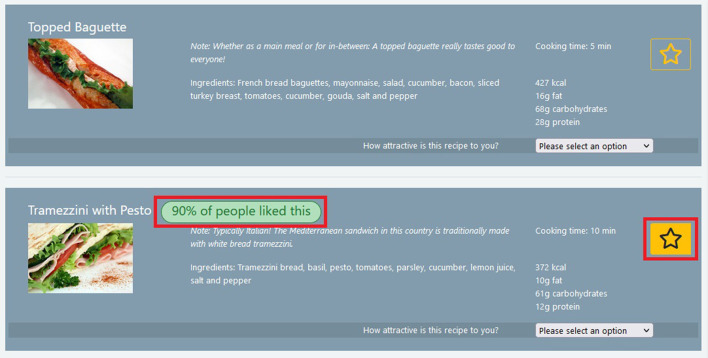
Items as displayed to participants, showing a non-nudged item at the top and the second item below showing our implementation of the hybrid nudge. The social information and the default setting are highlighted in red in the figure for illustration purpose; the red border was not present in the UI during the study. In the final study, the images and the displayed information were extracted from allrecipes.com, and the survey was administered via custom software.

Additionally, we were interested in testing the effectiveness of a combination of the default nudge and the social nudge. As shown in previous works (Lee et al., 2011; Hoenink et al., 2020), and as also mentioned above, the combination of nudges might be more effective than applying them individually. Other combinations of nudges than the tested one are of course possible as well. In this present work, we aim at gaining a first understanding of combined nudges, and we leave the exploration of other combinations for future work. An example for the user interface, which implements the hybrid nudge for the Sandwiches category, is shown in Figure 1.

The specifics of each nudge can be summarized as follows.

- Setting defaults: This is one of the most prominent digital nudges found in the literature (Weinmann et al., 2016; Mirsch et al., 2017). The nudge consists of pre-selecting a choice for subjects. This pre-selection can be in any position and does not necessarily need to be the first item in a list (Johnson et al., 2012) Several studies show that humans tend to stick to the pre-selected option much more often (Sunstein, 2014; Theocharous et al., 2019), although studies in restaurant settings show that participants sometimes tend to dodge healthy food defaults (Colby et al., 2020). In our application, one option was, therefore, already selected when the participant opened a page with choices.
- Highlighting: This nudge emphasizes the visual salience of options to increase the attention that is directed toward them (Caraban et al., 2019; Dai et al., 2020). Typically, this is done by changing the size and color of the text or increasing the contrast of options. In our study, we used a colored background behind the nudged option.
- Social Nudge: In situations of uncertainty, people tend to follow the lead of other like-minded people (Caraban et al., 2019). Some of the psychological phenomena described in the literature are that people follow the crowd, follow opinion-leaders or comply with social norms (Robinson et al., 2014; Mirsch et al., 2017; Caraban et al., 2019). By relying on one of these phenomena, we created a social nudge in the form of additional text above the nudged recipe. This text was the same for every participant and said: “90% of other people liked this.” We are aware that the term “liking” may be seen as being rather vague. Nonetheless, we chose to use this term as it is quite common on social media platforms and participants should, therefore, be able to interpret its meaning well in the given context.
- Hybrid (Default + Social): This hybrid represents a combination of two of the above nudges, which we tested to assess possible additive effects. For our study, we combined the methods of *setting defaults* and the *social nudge*.
- Warning: The warning nudge is different from the other nudges in our study because it does not aim to steer the user's decision *toward* a certain item, but to stimulate the user to *change* an initially made choice (Sunstein, 2014). In our case, we achieve this by providing specific information about the choice the user is about to make. When designing the warning nudge, we, therefore, selected one specific category for this nudge, in our case that of Desserts, and implemented the following logic. If the recipe chosen by the participant had a high amount of calories, a message was displayed to steer the participant toward a recipe with fewer calories. Similarly, if the recipe contained alcohol, the warning aimed to influence the decision toward ones that did not. The text of the warning read: “Please note that this dish contains alcohol/has a high amount of kilocalories per serving.”

To select categories and recipes for our study, we used the website *allrecipes.com*. We randomly selected five categories available on this website. Within these categories, we then selected six of the more popular recipes. Note that the number of options provided may affect the decision processes (e.g., choice overload) and the effectiveness of the nudges. Related studies suggest using six items to avoid the effects of choice overload (Bollen et al., 2010; Johns et al., 2013). A picture of each recipe and additional meta-data were gathered from the website and presented in the web application for each recipe.

#### 3.1.2. Participants

For the first study, we recruited 293 participants through the crowdsourcing platform Amazon Mechanical Turk. To increase the quality of the responses, we only allowed participants to complete our study who had a record of past good performance on the platform. Therefore, we used the “approval rate” as the performance measure, which had to be higher than 80%. This means that a participant had at least 80% of previous tasks completed satisfactorily. Furthermore, in order to deal with potentially unattentive participants, we implemented an *attention check* in the form of a question in the post-task questionnaire where one specific answer had to be selected^1^. Additionally, we removed participants who needed an unusually long time to make their decision, which we explain in more detail in section 5.1. After removing all potentially unreliable participants, we were left with 157 participants. Of these 157 participants, 79 were in the treatment group, and 78 were in the control group. The most frequent age group was “between 26 and 35,” and 90 were male (57%), and 67 were female (43%). Participants were paid 1$ for completing the task.

### 3.2. Results

Table 2 contains the results regarding the effectiveness of the used nudges. Each row represents a category and compares the results of the treatment and the control group. We show the used nudge for every category in the treatment group, the number of decisions, and how often the nudged item was selected. Since participants could freely choose the categories, we also report the number of decisions per category. Similarly, for the control group, we also include the number of decisions in every category. We do not have a nudged element in the control group. Therefore, we use the second recipe as a reference item and compare it to the (same) second item in the treatment group. This allows us to compare the rates at which the second recipe was selected in both designs and to thereby investigate the effectiveness of the used nudge. At the bottom row of the table, we provide the sums and the mean values.

**Table 2:** Effectiveness of all nudges in *Study-1* except for the warning nudge.

	**Treatment group**		**Control group**		
**Type of nudge (Category)**	***N***	**Nudged item selected**	***N***	**Target item selected**	**p-values**
Highlighting (Vegetarian)	52	14 (26.9%)	44	8 (18.2%)	0.57
Hybrid Nudge (Sandwiches)	58	34 (58.6%)	50	11 (22.0%)	**0.02**
Default (Pasta)	45	18 (40.0%)	51	14 (27.5%)	0.47
Social Nudge (Fish)	43	10 (23.3%)	40	5 (12.5%)	0.43
Overall	198	76 (36.8%)	185	38 (20.5%)	

*Choice frequencies of the target recipes (percentage in parentheses) in the treatment group (nudged) and control group (not nudged) by type of nudge. Column p-values refers to $X_{\left(df=1\right)}^{2}$ tests. Statistically significant differences are highlighted in bold*.

Table 2 shows that in all recipe categories, i.e., for all nudges that we implemented, the nudged item was selected by participants more frequently than the same second item was selected in the control group. The strongest increase was observed for the hybrid nudge, where 57.6% of participants selected the nudged item, whereas only 22.0% selected the same target item in the control group. Remember that assuming equal probabilities for each item to be selected in the control group, we would expect that each of the six items is selected in about 16.7% (= 1/6) of the cases. Overall, while the absolute numbers increased for all nudges, the observed differences were only statistically significant for the hybrid nudge in the Sandwiches category, as revealed by a Chi-Squared test [χ^2^_(1)_ = 5.2503, *p* = 0.02].

The results so far show that nudging *toward* a certain choice can be effective. We now draw our attention to the warning nudge that aims to drive participants away from their initial choice. Remember that for the Desserts category, a *warning* was shown immediately after the participants clicked on any item, informing them either that the chosen recipe contains alcohol or a high amount of calories. Thirty-nine participants chose the Desserts category as one of their three categories. We found that the warning nudge was highly effective. In 17 of these cases (44.4%), participants changed the selection after being shown the warning. Moreover, looking at the final selection, we observed that in about 75% of the cases, participants switched from an alcoholic dessert to one without alcohol or from a relatively high-calorie dessert to one with fewer calories.

*Study-1* aimed to explore if specific nudges work *in principle*, and we focused on a subset of nudges in specific food categories. Given that we did not implement a full factorial design between nudges and food categories, we cannot rule out that some nudges would work in other food categories, or that some categories may be easier to nudge than others. In any case, in our specific setting, only one nudge—the hybrid one—proved to change the participants' decision behavior effectively. Therefore, in *Study-2*, we assessed the effectiveness of the hybrid nudge systematically across four food categories.

## 4. Main Results: Study-2

### 4.1. Materials and Methods

#### 4.1.1. Study Design

With the results of *Study-1* in hand, our next aim was to validate that the effectiveness of the hybrid nudge is not limited to a particular category. Remember that the hybrid nudge was only applied in the Sandwiches category in *Study-1*. In *Study-2*, we, therefore, applied the hybrid nudge in all categories, except for Desserts, where we again used the negative warning nudge.

A minor modification was made to the social nudge in *Study-2* to increase the realism of the setting. In *Study-1* the social nudge was implemented by showing to participants for the nudged recipe that “*90% of other people liked this*.” Since we show this nudge for all categories except the Desserts in *Study-2*, participants might be surprised to see exactly 90% as a value for all their chosen categories. Therefore, we randomly varied the displayed percentage to lie between 90 and 94%.

#### 4.1.2. Participants

We conducted the second study with 60 participants, who were recruited on Amazon Mechanical Turk in the same way as we did for *Study-1* participants, using a minimum approval rate of 80%. We again included an attention check and removed the outliers, leaving us with 49 participants for this study. All participants were part of the additional treatment group, showing the hybrid nudges in all categories except Desserts. In *Study-2*, 37 participants were male (76%), and 12 participants were female (24%). The age group “between 26 and 35” was the most frequent one.

The participants in *Study-2* were similar to those of *Study-1* in terms of age and gender. As can be seen from the results of the post-task questionnaire, which can be found in Table A1 in the Supplementary Data, the ratings regarding the importance of healthy eating, cooking skills, and the interest in nutritional facts did not differ significantly between the groups. Therefore, these person characteristics are unlikely responsible for the differences between the control and treatment groups. Moreover, participants in both studies perceived the decision task similarly in both studies. Given this similarity of the conditions, we compare the treatment group with the control group from *Study-1*.

### 4.2. Results

Table 3 shows our results regarding the effectiveness of the nudges in *Study-2*, organized in the same way as for *Study-1*. This time, we found that the hybrid nudge was effective in all tested categories. Moreover, the differences to the control group are statistically significant for all cases according to Chi-Squared tests.

**Table 3:** Effectiveness of all nudges in *Study-2* except for the warning nudge.

	**Treatment Group**		**Control Group**		
**Type of Nudge (Category)**	**N**	**Nudged Item Selected**	**N**	**Target Item Selected**	**p-values**
Hybrid Nudge (Vegetarian)	27	15 (55.6%)	44	8 (18.2%)	**0.042**
Hybrid Nudge (Sandwiches)	30	19 (63.3%)	50	11 (22.0%)	**0.027**
Hybrid Nudge (Pasta)	29	21 (72.4%)	51	14 (27.5%)	**0.031**
Hybrid Nudge (Fish)	35	18 (51.4%)	40	5 (12,5%)	**0.015**
Overall	121	73 (60.3%)	185	38 (20.5%)	

*Choice frequencies of the target recipes (percentage in parentheses) in the treatment group (nudged) and control group (not nudged) by type of nudge. Column p-values refers to $X_{\left(df=1\right)}^{2}$ tests. Statistically significant differences are highlighted in bold*.

For the category of Sandwiches, for which we found the hybrid nudge to be effective already in *Study-1*, it turned out that the percentage of participants who selected the nudged sandwich was in the same range and even slightly higher in *Study-2*. On average, and across all categories, the nudged item was selected in more than half (60.3%) of the cases. This clearly speaks for the effectiveness of the hybrid nudge. Again, remember that the likelihood of the nudged item to be chosen would be only 16.6% if we assume that each item will be chosen with equal probability.

The negative warning nudge was again highly effective, and around 37% of the participants changed their minds after being shown the warning. This rate is well aligned with the previous observations from *Study-1* and confirms the effectiveness of the warning nudge.

## 5. Effects on the Decision Process

Having discussed our main results regarding the effectiveness of digital nudges for food choices, we now present the results of additional analyses that we performed to understand better how digital nudges work.

### 5.1. Decision Times

One of our research questions raised in section 1 was if participants would be more efficient in making decisions when being nudged. Indications that nudges could lead to a faster decision process were previously reported by Mirsch et al. (2017) and Caraban et al. (2019). We, therefore, investigated how long participants needed to choose a recipe under the different conditions (with and without nudges). For our analysis, we relied on the timestamps that were automatically logged by the application used for our studies.

Before running the analysis, we inspected the data and found indications of outliers, which we removed to avoid that our results are distorted, e.g., by participants who took excessively long to complete the task. Our approach to removing these outliers was to use three times the interquartile range as a threshold for the removal (Pollet and van der Meij, 2017). With this approach, we removed nine participants from the control group in *Study-1*, eleven from the treatment group of *Study-1*, and three from *Study-2*. Table 4 shows the resulting mean decision times and standard deviations for each group. A closer inspection of the decision times and distributions revealed that the data was skewed to the left for each group (control group: 1.82; *Study-1*: 2.26; *Study-2*: 1.34). We, therefore, log-transformed the decision times to be able to apply our statistical tests reliably.

**Table 4:** Mean decision times in seconds by group (*SD* in parentheses).

**Group**	**N**	**Mean decision time**
*Control*	185	75.8 (50.2)
*Study-1 Treatment*	201	59.5 (43.5)
*Study-2 Treatment*	121	62.7 (38.7)

*The reported statistics include the highlighting, default, hybrid, and social information nudge*.

For our first analysis, we focus on the first four nudging types (highlighting, default, hybrid, and social information), as they do not introduce an additional step to the decision as the warning nudge does. Remember that the warning nudge is likely to increase the decision time as additional information is displayed after the participants' initial selection. Therefore, we put it aside in this analysis. For the remaining four nudges that steer users toward a certain selection, we observe that the mean decision time for the treatment groups in *Study-1* and *Study-2* is around 13 s (around 17%) shorter than in the control group. In order to see if this reduction in the decision times was also significant, we performed a repeated ANOVA. The repeated ANOVA using the log-transformed decision time revealed a significant difference between the groups [*F*_(2,200)_ = 5.016, *p* < 0.01]. A *post-hoc* comparison using Tukey HSD then confirmed that participants in the treatment groups of *Study-1* (*p* < 0.001) and *Study-2* (*p* = 0.03) needed significantly less time than the control group. Therefore, we conclude that digital nudges, as explored in our studies, can be a suitable means to increase decision efficiency.

Having established that participants were faster in both treatment groups when only considering the nudges toward a certain option, we next investigate how the *individual nudges* affected the decision times. The mean and standard deviations for the individual nudges and the relative change to the control group are shown in Table 5. On average, participants who saw the highlighting, default, social information, and the hybrid nudge took less time than the control group. In contrast, the warning nudge slightly increased the time participants needed to make a decision. This last finding is not surprising, because in the warning nudge condition participants were shown additional information that they had to process, and many participants then also reconsidered their initial choices. Furthermore, we see that the decision time decreases from category to category. Remember that the categories and their order were static and thus the reduction may be due to familiarization with the display.

**Table 5:** Mean and standard deviation for decision times (in seconds) by nudge type and food category.

**Group**	**Type of nudge**	**Food category**	**Mean decision time**	**Relative change to control**	**p-value**
*Control*					
	None	Vegetarian	103.0 (62.0)	–	–
	None	Sandwiches	80.3 (49.3)	–	–
	None	Pasta	65.0 (40.2)	–	–
	None	Fish	54.3 (32.3)	–	–
	None	Desserts	52.4 (26.1)	–	–
*Study-1 Treatment*					
	Highlighting	Vegetarian	**85.9 (61.9)**	**–16.6**%	**0.05**
	Hybrid	Sandwiches	**53.2 (31.9)**	**–33.7%**	**<0.001**
	Default	Pasta	**54.9 (31.4)**	**–15.6%**	**0.02**
	Social information	Fish	46.9 (26.9)	–13.6%	0.38
	Warning	Desserts	60.4 (26.9)	+15.3%	0.10
*Study-2 Treatment*					
	Hybrid	Vegetarian	81.9 (45.9)	–20.5%	0.10
	Hybrid	Sandwiches	77.7 (45.4)	–3.2%	0.83
	Hybrid	Pasta	47.0 (16.4)	–27.7%	0.07
	Hybrid	Fish	48.1 (27.9)	–11.4%	0.44
	Warning	Desserts	59.1 (14.8)	+12.8%	0.07

*Means and standard deviations are given in the form of M(SD). Additionally, we provide the relative change to the control group and p-values for the t-tests. Statistically significant differences are highlighted in bold*.

We conducted *t*-tests further to analyze the differences between the log-transformed decision times. The results of these *t*-tests are shown in Table 5 in the last column. Based on these results we see three significant differences. The first one is between the highlighting nudge in the Vegetarian category in *Study-1* and the control group, *t*_(94)_ = −1.9521, *p* = 0.05. We also see that the hybrid nudge applied in *Study-1* in the Sandwiches category reduced the overall decision time of participants, *t*_(106)_ = −3.46, *p* < 0.001. Lastly, in *Study-1*, we see another significant reduction, *t*_(94)_ = −2.37, *p* = 0.02, for the default nudge in the Pasta category and the control group. Therefore, we conclude that certain nudges have the potential to reduce the required decision time of online food selection scenarios. A reduction of the decision time may also lead to a reduction of the perceived decision effort, as these two aspects are closely related. The warning nudge, as expected, led to longer decision times compared to the control group, but the increase was not statistically significant. Even though participants had to change their decision, which meant that they had to consider most options again, they reconsidered their selection and choosing, in most cases (around 75%), a preferable recipe. Although more research is needed to investigate nudges that pro-actively intervene with the decision, based on our findings, we recommend using the warning nudge as an effective means to change the decision behavior without negative effects on decision times and decision effort.

Although the hybrid nudge led to a reduced decision time in the treatment group of *Study-1*, we found no significant reduction of decision times in any treatment group of *Study-2*. A possible explanation for the absence of any significant effect on the decision times in *Study-2* could be the smaller group of participants in *Study-2* and the resulting small number of decisions in each food category. Nonetheless, we see an overall reduction of the decision time for participants in the treatment group of *Study-2*, see Table 4.

### 5.2. Analysis of Post-choice Responses

Remember that participants were asked a number of questions after each of the three choices. These questions should inform us about different aspects of the decision process and to what extent the nudges affect these aspects. One underlying hypothesis, for example, is that using a nudge will make it easier for people to make a satisfactory decision (Mirsch et al., 2017). Therefore, after each choice, we asked participants six questions revolving around choice difficulty and choice satisfaction, which they had to answer on a 7-point Likert scale (1-strongly disagree, 4-neither agree nor disagree, 7-strongly agree). Furthermore, the participants were asked to provide a free text answer on *why* they selected a certain recipe.

#### 5.2.1. Questionnaire Results

We first examined the participants' responses to the six questions. Table 6 shows all questions with the corresponding factor that was investigated with that question.

**Table 6:** Questions of the post-choice questionnaire with the corresponding abbreviation and factor.

**Factor**	**Question**
Difficulty	It was difficult for me to choose one recipe
Satisfaction	I am satisfied with my selection
Confidence	I am confident I made the best possible decision in this category
Navigation	It was easy to choose the most delicious recipe in this category
Belief	I am convinced the chosen recipe suits my taste best
Repeated selection	I would choose the same recipe again

The means and standard deviations for the responses to the questions are shown in Table A2 in the Supplementary Data section. The main findings can be summarized as follows^2^ Participants in the two treatment groups and the control group showed no differences in perceptions of difficulty, satisfaction, navigation, belief, and the likelihood of selecting the same recipe again after making their decision. As for confidence we identified no difference for the treatment group in *Study-1* but we saw a significant increase, (*U* = 541, *p* = 0.03), between the treatment group of *Study-2* (*M* = 5.93, *SD* = 1.10) and the control group (*M* = 5.51, *SD* = 1.24). Although nudges caused participants to select the nudged item more often and therefore steer their behavior, they did not negatively change the way participants evaluated the decision situation. Based on this result, we can suggest that nudges can be applied without concern to our investigated factors. Furthermore, we see that nudges might have the potential to increase the confidence in a selected recipe and improve the decision-making for participants.

#### 5.2.2. Analysis of Free Text Answers

As the last step, the free text answers of the questionnaire were analyzed. Remember that participants were asked here to explain the reasons for their choices for each recipe. We followed an inductive coding process and card sorting approach to categorize the given explanations. Based on the given answers, we extracted keywords and grouped them into categories. The resulting eight categories are shown in Table 7 with the corresponding total number of mentions and the percentage of mentions concerning all answers measured across that group. Note that an individual answer can be assigned to multiple categories. An example of a statement that falls into two categories was “*I liked how the dish looked and thought it must taste delicious*..” This phrase was categorized both under “Visual Characteristics” and under “Taste”.

**Table 7:** Categorized free text explanations after each decision.

	**Preferred Food**	**Visual characteristics**	**Convenience**	**Ingredients**	**Taste**	**Experience**	**Recommended**	**Other**	**All**
Control	106 (34%)	17 (5%)	14 (5%)	31 (10%)	67 (22%)	13 (4%)	0 (0%)	62 (20%)	310
Study 1	109 (34%)	35 (11%)	12 (4%)	35 (11%)	60 (19%)	3 (1%)	8 (3%)	44 (14%)	317
Study 2	51 (27%)	22 (12%)	10 (5%)	31 (17%)	30 (16%)	5 (3%)	15 (8%)	23 (12%)	187

*Numbers indicate how often a free-text answer was assigned to a certain category*.

Table 7 shows the outcome of this process. While the numbers are often quite similar across different groups (*Control* and *Treatment* of *Study-1, Study-2*), there are two notable exceptions. The first exception is that participants in the control group did not refer as much to visual characteristics in their explanations as to the treatment groups. This might indicate that the nudges applied in the treatment group diverted participants' attention from the recipe pictures to the nudges. For the second exception, we find that in particular in *Study-2*, participants much more often use explanations of the category “Recommended” than in the control group. Since the hybrid nudge that was used in *Study-2* includes a (social) recommendation, this might indicate that the participants actually take the information conveyed through the nudge into account when making their decisions.

While participants still recognized that they were influenced, this influence had no immediate negative impact on their perception of their decision. Loewenstein et al. (2015) showed that informing participants about a nudge (i.e., default nudge) did not change the effectiveness of the nudge. Although not under identical treatments, our results extend these findings. They indicate that while some participants recognize that they are being nudged, this might not harm their perception of the decision in terms of our observed factors.

## 6. Discussion

Our results clearly confirm that digital nudges can be an effective means to influence the choice behavior of online users in a food-related decision scenario. First, we found that nudges can be *effective*, both in terms of steering online users to a certain choice and also in terms of guiding them away from a certain choice (through the warning nudge). As a result, we see digital nudging as a promising approach to influence the cooking and eating behavior of online users toward healthier choices. However, our studies also reveal that the type of the nudge can matter. According to our experiments, only a combination of two nudging principles—highlighting and providing social information—consistently led to the desired effects across different food categories. Future studies could improve our understanding of how hybrid nudges work in the food domain by relating the effects on choices, decision time, and other process variables reflecting the decision experience to the specific nudging mechanisms implemented in the hybrid. From a practical perspective this means the system and user interface designers should consider and explore various types of individual and combined nudges for their particular application setting.

Another main result of our studies is that digital nudges can also help to increase the *efficiency* of the decision-making process, notably without a decrease in choice satisfaction and choice confidence. Notably, even the hybrid nudge, which conveyed additional information to be processed by the participants, led to a significant reduction of decision time. This finding is consistent with the idea that nudges may often promote heuristic, faster decision-making, and reduce cognitive effort. Also, the fact that satisfaction and choice confidence did not differ between nudged and not nudged participants is promising, given the potentially negative reactions to being influenced or manipulated. Our findings are thus in line with those showing relatively high public approval for nudge interventions in the health domain (Krisam et al., 2021).

We carried out further analyses, but we do not present them in detail here in order to keep the work focused on our main research questions. One example for such an analysis is gender-specific differences, which have only started to investigate in this study. In this context, we were able to identify that there seem to be differences in terms of how male and female participants experience the underlying choice problem. We found such gender-related differences, in particular for the control group, where no nudges were displayed. To some extent, digital nudges seem to have led to a reduction of the differences, e.g., when for males, the level of choice satisfaction and choice confidence increased in the presence of nudges.^3^ More research is however still needed regarding gender-specific differences, as well as regarding other personal characteristics such as dietary requirements, food preferences or attitudes toward environmentally sustainable food consumption (Vermeir et al., 2020).

In terms of research limitations and threats to validity, we are aware that conducting studies of this type with crowdworkers who are paid for the task may have certain risks. Therefore, in our studies, we have taken measures to ensure that only responses by attentive and experienced crowdworkers were considered. Furthermore, since the participants, on average, considered their interest in healthy food and their cooking skills fairly high, we are confident that the user population of our study is representative of a certain segment of users of online recipe sites. Generally, another possible limitation of our study may also lie in the selection of a relatively small number of recipes based on their popularity at that time. In order to validate the generalizability of our results, an approach as proposed by Elsweiler et al. (2017)—who used similarity metrics to select recipes—could be applied. Furthermore, additional studies may be needed, for example, to investigate potential effects of the comparability of recipes within a consideration set.

So far, our studies have focused on a handful of different ways to nudge participants. While the examined nudges comprise some of the most widely analyzed ones in the research literature, the design space for nudges is large, both in terms of the underlying psychological phenomena and the particular implementation in the user interface, see (Jesse and Jannach, 2021). More research is therefore required to understand if other nudges are even more effective and to what extent the effectiveness of a nudge is tied to a particular choice problem. Nonetheless, we see our work as providing important additional evidence regarding the effectiveness of digital nudging. Furthermore, our work highlights that the specifics of how the nudge is designed may matter and must be kept in mind when implementing digital nudges.

## Data Availability Statement

The datasets presented in this study can be found in online repositories. The names of the repository/repositories and accession number(s) can be found below: https://osf.io/ketf3/.

## Ethics Statement

Ethical review and approval was not required for the study on human participants in accordance with the local legislation and institutional requirements. The patients/participants provided their written informed consent to participate in this study. Written informed consent was obtained from the individual(s) for the publication of any potentially identifiable images or data included in this article.

## Author Contributions

MJ: conceptualization, software, formal analysis, investigation, and writing. DJ: conceptualization, methodology, and writing. BG: conceptualization, methodology, and formal analysis. All authors contributed to the article and approved the submitted version.

## Funding

MJ is supported through the doctoral school “DECIDE,” funded by the University of Klagenfurt.

## Conflict of Interest

The authors declare that the research was conducted in the absence of any commercial or financial relationships that could be construed as a potential conflict of interest.

## Publisher's Note

All claims expressed in this article are solely those of the authors and do not necessarily represent those of their affiliated organizations, or those of the publisher, the editors and the reviewers. Any product that may be evaluated in this article, or claim that may be made by its manufacturer, is not guaranteed or endorsed by the publisher.

## Footnotes

^1^The text used for the attention check was: “I am answering with care and choose option 2 for this question”.

^2^We conducted Mann-Whitney U tests to check the observed differences for statistical significance since the underlying data are ordinally scaled.

^3^We share a spreadsheet with our initial gender-specific analysis online.

## Appendix

### Supplementary Data

For reproducibility, the data collected in the studies and the scripts that were used for the data analysis can be found online at https://osf.io/ketf3/.

**Table A1:** Mean answer given in the final questionnaire decision.

**Question**	**Control group** **(***N*** = 78)**	**Study-1** **(***N*** = 79)**	**Study-2** **(***N*** = 49)**
*Q1:* I am confident I will like the chosen dish	5.95 (1.12)	5.87 (0.99)	5.86 (1.03)
*Q2:* Choosing my favorite dishes was easy	5.58 (1.09)	5.62 (1.33)	5.76 (1.16)
*Q3:* I would like to try the chosen recipes	5.71 (1.21)	5.53 (1.13)	5.96 (0.99)
*Q4:* The amount of information available was sufficient to make good decisions	5.71 (1.07)	5.71 (1.07)	5.92 (0.78)
*Q5:* Eating healthy is important to me	5.58 (1.38)	5.70 (1.13)	5.67 (1.26)
*Q6:* I am interested in the nutritional facts of the meals I prepare	5.68 (1.26)	5.56 (1.11)	5.71 (1.36)
*Q7:* I rate my cooking skills high	5.35 (1.42)	5.48 (1.27)	5.61 (1.26)
*Q8:* The system guided me through the decision making process	5.63 (1.18)	5.53 (1.06)	5.65 (1.12)

*The mean and standard deviation to each question are in the form of M(SD)*.

**Table A2:** Mean answer given in the questionnaire after each decision split by type of nudge and food category.

**Group**	**Type of nudge**	**Food Category**	**N**	**Difficulty**	**Satisfaction**	**Confidence**	**Navigation**	**Belief**	**Repeated selection**
*Control*									
	None	Vegetarian	44	4.58 (1.95)	5.89 (1.13)	5.73 (1.04)	5.36 (1.50)	5.91 (1.20)	5.82 (1.23)
	None	Sandwiches	50	4.54 (1.85)	6.14 (0.78)	5.84 (1.08)	5.66 (1.29)	5.90 (1.04)	5.80 (1.12)
	None	Pasta	51	4.39 (1.96)	5.71 (1.19)	5.51 (1.24)	5.37 (1.22)	5.75 (1.18)	5.63 (1.13)
	None	Fish	41	4.83 (1.70)	5.80 (1.23)	5.82 (1.45)	5.73 (1.38)	5.66 (1.49)	5.76 (1.26)
	None	Desserts	48	4.58 (1.90)	5.92 (1.05)	5.81 (1.04)	5.23 (1.55)	5.79 (1.09)	5.96 (1.18)
*Study-1*									
	Highlighting	Vegetarian	52	4.63 (1.82)	5.98 (1.24)	5.98 (0.85)	5.65 (1.14)	5.63 (1.17)	5.71 (1.09)
	Hybrid	Sandwiches	58	4.57 (1.80)	6.00 (0.82)	5.76 (1.14)	5.47 (1.33)	5.71 (1.14)	5.67 (1.22)
	Default	Pasta	45	4.13 (1.77)	6.04 (0.85)	5.96 (1.04)	5.64 (1.19)	5.80 (1.06)	5.91 (0.95)
	Social Information	Fish	43	4.56 (2.03)	5.88 (1.10)	5.91 (1.02)	5.40 (1.47)	5.79 (1.04)	5.98 (1.20)
	Warning	Desserts	39	4.33 (1.96)	6.15 (1.04)	5.82 (1.02)	5.36 (1.35)	5.64 (0.99)	6.10 (0.79)
*Study-2*									
	Hybrid	Vegetarian	27	4.37 (1.86)	6.26 (0.98)	5.89 (1.12)	5.48 (1.34)	5.78 (1.31)	5.70 (1.32)
	Hybrid	Sandwiches	30	3.77 (2.14)	6.13 (0.78)	6.33 (0.88)	5.60 (1.22)	6.07 (0.98)	6.07 (1.05)
	Hybrid	Pasta	29	4.14 (2.13)	5.93 (0.96)	**5.93 (1.10)** **↑**	5.79 (1.18)	6.00 (1.31)	6.03 (1.02)
	Hybrid	Fish	35	4.71 (1.67)	6.06 (1.00)	5.89 (1.02)	5.40 (1.52)	5.89 (0.90)	5.71 (1.43)
	Warning	Desserts	26	4.50 (2.06)	6.23 (0.77)	6.04 (1.08)	5.50 (1.24)	5.77 (1.18)	6.08 (0.89)

*The mean and standard deviation to each question are in the form of M(SD). Significant differences are highlighted and marked with an arrow to indicate the direction of the difference. See Table 6 for the wording of questions*.
